# A Versatile Overexpression Strategy in the Pathogenic Yeast *Candida albicans*: Identification of Regulators of Morphogenesis and Fitness

**DOI:** 10.1371/journal.pone.0045912

**Published:** 2012-09-25

**Authors:** Murielle Chauvel, Audrey Nesseir, Vitor Cabral, Sadri Znaidi, Sophie Goyard, Sophie Bachellier-Bassi, Arnaud Firon, Mélanie Legrand, Dorothée Diogo, Claire Naulleau, Tristan Rossignol, Christophe d’Enfert

**Affiliations:** 1 Institut Pasteur, Unité Biologie et Pathogénicité Fongiques, Département Génomes et Génétique, Paris, France; 2 INRA, USC2019, Paris, France; 3 Université Paris Diderot, Sorbonne Paris Cité, Cellule Pasteur, Paris, France; New Jersey Medical School, University of Medicine and Dentistry of New Jersey, United States of America

## Abstract

*Candida albicans* is the most frequently encountered human fungal pathogen, causing both superficial infections and life-threatening systemic diseases. Functional genomic studies performed in this organism have mainly used knock-out mutants and extensive collections of overexpression mutants are still lacking. Here, we report the development of a first generation *C. albicans* ORFeome, the improvement of overexpression systems and the construction of two new libraries of *C. albicans* strains overexpressing genes for components of signaling networks, in particular protein kinases, protein phosphatases and transcription factors. As a proof of concept, we screened these collections for genes whose overexpression impacts morphogenesis or growth rates in *C. albicans*. Our screens identified genes previously described for their role in these biological processes, demonstrating the functionality of our strategy, as well as genes that have not been previously associated to these processes. This article emphasizes the potential of systematic overexpression strategies to improve our knowledge of regulatory networks in *C. albicans*. The *C. albicans* plasmid and strain collections described here are available at the Fungal Genetics Stock Center. Their extension to a genome-wide scale will represent important resources for the *C. albicans* community.

## Introduction

*Candida albicans* is a normal member of human natural cavities, especially of the gastrointestinal and urogenital tracts [1], [2]. In addition to its commensal activity and under specific conditions, this yeast becomes one of the major invasive fungal pathogen of humans, and can cause both mucosal and life-threatening disseminated infections [3], [4]. The significant mortality rate associated with candidiasis in immunocompromised patients drives the research efforts to improve our knowledge of *C. albicans* biology and pathogenesis [5].

During the last decade, progresses in gene inactivation methodologies have been the driving force to characterize *C. albicans* molecular processes [6], [7]. Several collections of heterozygous and homozygous knock-out (KO) mutants have been generated. These resources are now invaluable to study *C. albicans* regulatory networks, virulence, hyphal morphogenesis, biofilm formation, identify drug targets and evaluate the mode-of-action of antifungal compounds [8]–[20]. However, the use of KO mutants shows some limitations. First, since *C. albicans* is an obligate diploid organism with no known meiotic cycle, two rounds of gene disruption are required to produce each deletion mutant. The pioneering works for the construction of homozygous KO mutants in the *C. albicans* genome mentioned above were indeed tedious and time-consuming. Second, gene deletion approaches are not optimal in the case of functional redundancy or essential genes. To date, less than 25% of *C. albicans* genes have been functionally characterized, indicating that new approaches must be developed for the study of this pathogen.

The use of both systematic KO and overexpression (OE) approaches to investigate cellular processes has proven highly successful in the model yeast *Saccharomyces cerevisiae*. Genome-wide collections of *S. cerevisiae* OE strains have been assembled and used to perform large-scale functional analyses, leading to the identification of new signalling pathways, new targets and functions for transcription factors or protein kinases and, more largely, to improve our image of the functional landscape of the cell [21]–[27]. In contrast, OE strategies have not been exploited extensively in *C. albicans*. Fu *et al*. [28] established a collection of 26 heterozygous OE strains for genes encoding glycophosphatidylinositol-anchored (GPI) proteins. This study demonstrated the role in adherence for the product of the *IFF4* gene, one of 11 members of the IFF genes family. More recently, Sahni *et al.* [29] constructed an OE library of 103 transcription factors which has been used in two independent screens, demonstrating a role for the Tec1 transcription factor in the response of white cells to pheromones [29] and identifying a critical function for the Brg1 transcription factor in *C. albicans* biofilm formation, filamentous growth and virulence [30]. Other studies have used OE of selected genes in order to test the relevance of regulatory networks and functional pathways inferred from gene expression studies [31]–[34]. Nevertheless, the collections of OE strains yet available are focused and despite the encouraging results obtained in the studies mentioned above, a flexible collection of OE plasmids encompassing the 6200 *C. albicans* genes and the corresponding *C. albicans* OE strains are still lacking.

Here, we constructed two collections of *C. albicans* OE strains, enriched for genes encoding protein kinases, protein phosphatases, transcription factors and other signalling proteins. These new resources took advantage of the highly efficient Gateway® technology to provide versatility to our system, leading to a first generation *C. albicans* ORFeome. To test the functionality and applications of our strategy, we performed screens for regulators of morphogenesis and growth rate in *C. albicans*, two research areas of crucial interest for the development of new antifungal strategies. Our results highlight the value of using gene OE as a complement to gene inactivation to both uncover gene function and reveal new regulators in *C. albicans*. They also pave the way for the development of genome-wide OE approaches for this major pathogen.

## Results and Discussion

### Development of Gateway Vectors for Overexpression in *Candida albicans*

**Figure 1:**
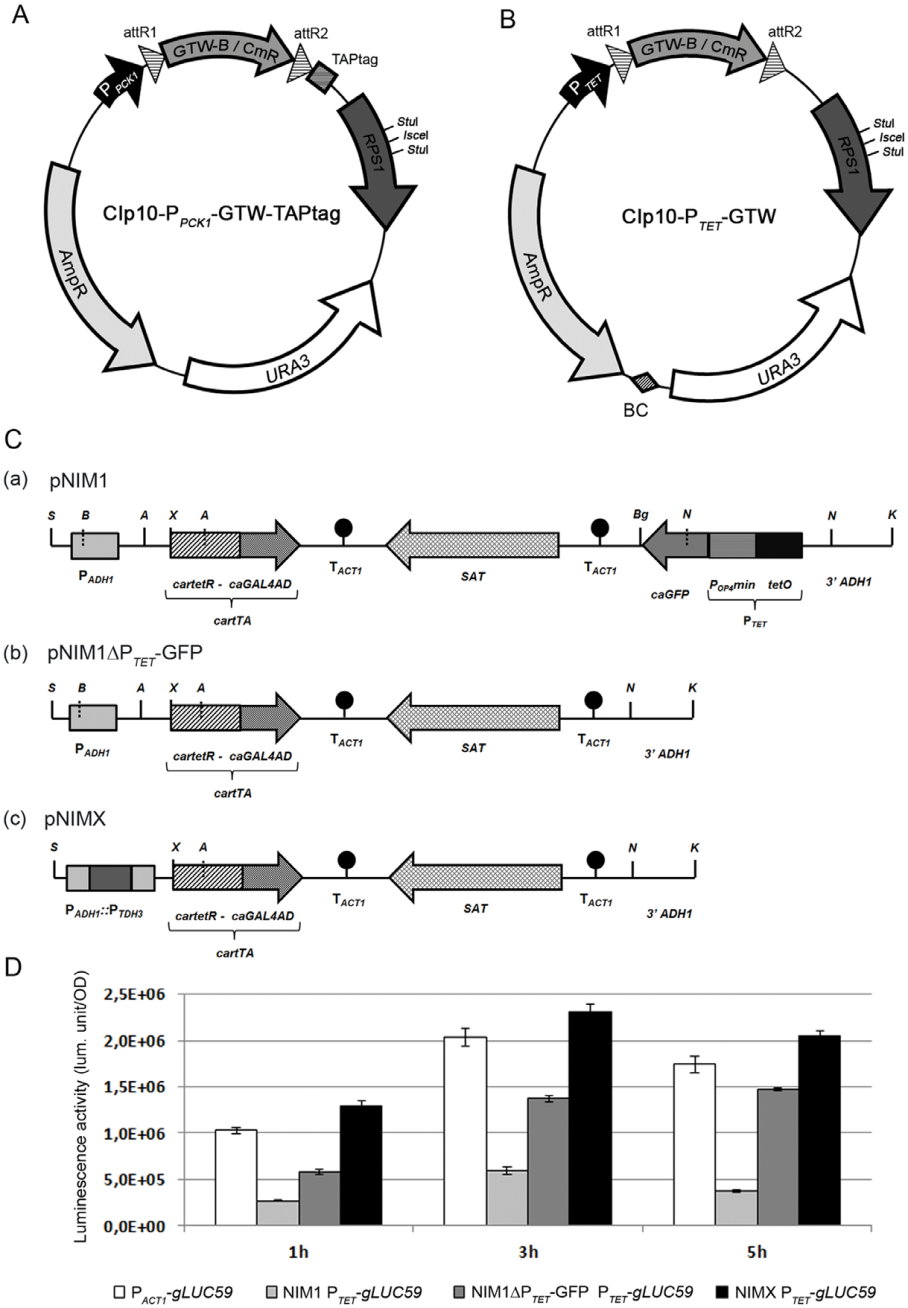
Gateway-adapted OE systems for *C. albicans*. Schematic maps of the CIp10-P*_PCK1_*-GTW-TAPtag (A) and CIp10-P*_TET_*-GTW (B) vectors. The presence of *attR* recombination sites allows Gateway®-mediated cloning of ORFs in place of the GTW-B/CmR cassette. ORFs are expressed from the *PCK1* promoter (P*_PCK1_* **- A**) or the *TET* promoter (P*_TET_* - **B**) that are induced in gluconeogenic growth conditions or in the presence of tetracycline derivatives (doxycycline, anhydrotetracycline), respectively. In the first case, ORFs are fused to a TAPtag coding region, thus allowing production of proteins TAPtagged at their C-terminus. In the second case, each ORF is associated to a unique barcode (BC). Derivatives of CIp10-P*_PCK1_*-GTW-TAPtag and CIp10-P*_TET_*-GTW can be targeted to the *C. albicans RPS1* locus when linearized with *Stu*I or *I-Sce*I and *C. albicans* transformants are selected for uridine prototrophy conferred by the *URA3* gene. **C. Schematic maps of the different transactivation cassettes used to promote expression from the** ***TET*** **promoter.** The pNIMX cassette (**c**) is a derivative of pNIM1 (**a**; [39]). pNIMX was generated by deleting the P*_TET_*-GFP fusion in pNIM1, yielding pNIM1ΔP*_TET_*-GFP (**b**), and subsequently exchanging the P*_ADH1_* promoter upstream of the *cartTA* region by the *TDH3* promoter (P*_TDH3_*). Relevant restriction sites are shown: A: *Acl*I, B: *Bam*HI, Bg: *Bgl*II, K: *Kpn*I, N: *Nco*I, S: *Sac*II, X: *Xba*I. **D. The pNIMX transactivator cassette provides enhanced P** ***_TET_*** **-driven OE.** *C. albicans* strains harbouring the CIp10-P*_TET_*-*gLUC59* plasmid, with the *gLUC59* luciferase reporter gene under the control of P*_TET_*, and either pNIM1, pNIM1ΔP*_TET_*-GFP or pNIMX (CEC1909, CEC2249 or CEC3083 respectively) were grown in YPD liquid medium supplemented with 50 µg.mL**^−^** ^1^ Dox. A *C. albicans* strain harbouring CIp10-P*_ACT1_*-*gLUC59* plasmid (CEC988) and expressing the *gLUC59* reporter gene constitutively was used as a control and grown in the same conditions. Data represent luciferase specific activity detected from the different strains after 1, 5 and 8 h of growth in the presence of Dox. Assays were performed in duplicate and means and SD are shown.

In order to develop a collection of *C. albicans* OE strains, we have taken advantage of the Gateway® methodology that enables recombination-mediated cloning of PCR-amplified ORFs into a donor vector and their subsequent recombination-mediated transfer into a variety of customized destination vectors [35]. We developed two conditional OE destination vectors named CIp10-P*_PCK1_*-GTW-TAPtag and CIp10-P*_TET_*-GTW (Fig. 1A and 1B respectively), both being derivatives of the *C. albicans* CIp10 integrative vector [36]. CIp10-P*_PCK1_*-GTW-TAPtag carries a Gateway® cassette flanked by the gluconeogenesis-induced *C. albicans PCK1* promoter (P*_PCK1_*; [37]) and an in-frame sequence encoding a tag for tandem-affinity purification (TAPtag; Fig. 1B; [38]). Expression from P*_PCK1_* is achieved in the presence of casamino acids and repressed in the presence of glucose. CIp10-P*_TET_*-GTW contains the *TET* promoter (P*_TET_*; [39]) that is activated in the presence of tetracycline derivatives. In contrast to CIp10-P*_PCK1_*-GTW-TAPtag, CIp10-P*_TET_*-GTW is not equipped with a TAPtag but with a unique barcode system (Fig. 1B and Materials and Methods for the barcoding procedure).

### Optimisation of Tetracycline-dependent Overexpression in *Candida albicans*

Expression from the P*_TET_* promoter requires a *C. albicans*-adapted reverse Tet-dependent transactivator (*cartTA*) that binds the *tetO* sequences in the P*_TET_* promoter in a tetracycline-dependent manner and drives transcription through the activation domain of the Gal4 protein of *S. cerevisiae* [39]. Different plasmids allowing expression of *cartTA* in *C. albicans* are available among which pNIM1 whereby *cartTA* is expressed from the promoter of the *ADH1* gene and that harbours a P*_TET_*-GFP fusion (Fig. 1C–a; [39]). We reasoned that tetracycline-dependent OE of genes cloned downstream of P*_TET_* on CIp10-P*_TET_*-GTW plasmids might be enhanced by removing the P*_TET_*-GFP fusion from pNIM1 and expressing *cartTA* from a stronger promoter than that of the *ADH1* gene. Therefore we produced two derivatives of the pNIM1 plasmid: pNIM1ΔP*_TET_*-GFP (Fig. 1C–b) lacks the P*_TET_*-GFP fusion; pNIMX (Fig. 1C–c) lacks this fusion and carries the *cartTA* coding region placed under the control of the strong and constitutive *C. albicans TDH3* promoter (P*_TDH3_*; [40]. In order to test the relative efficiency of the pNIM1, pNIM1ΔP*_TET_*-GFP and pNIMX plamsids plasmids at driving OE from the P*_TET_* promoter, these plasmids were introduced in a *C. albicans* strain that harboured a fusion between P*_TET_* and the *gLUC59* luciferase reporter gene [41]. Results presented in Fig. 1D showed that luciferase levels achieved from the strains harbouring pNIM1ΔP*_TET_*-GFP or pNIMX were respectively 3 or 5 times higher than those obtained in a *C. albicans* strain harbouring pNIM1. Noticeably, luciferase levels achieved from the strain transformed with pNIMX and the P*_TET_-gLUC59* fusion were above those observed in a *C. albicans* strain harbouring a P*_ACT1_-gLUC59* fusion (Fig. 1D). Thus, *C. albicans* strains harbouring pNIMX were subsequently used to drive expression from the P*_TET_* promoter.

### Validation of the OE-Gateway Vectors Developed for *Candida albicans* and Quantification of the OE Level

**Figure 2:**
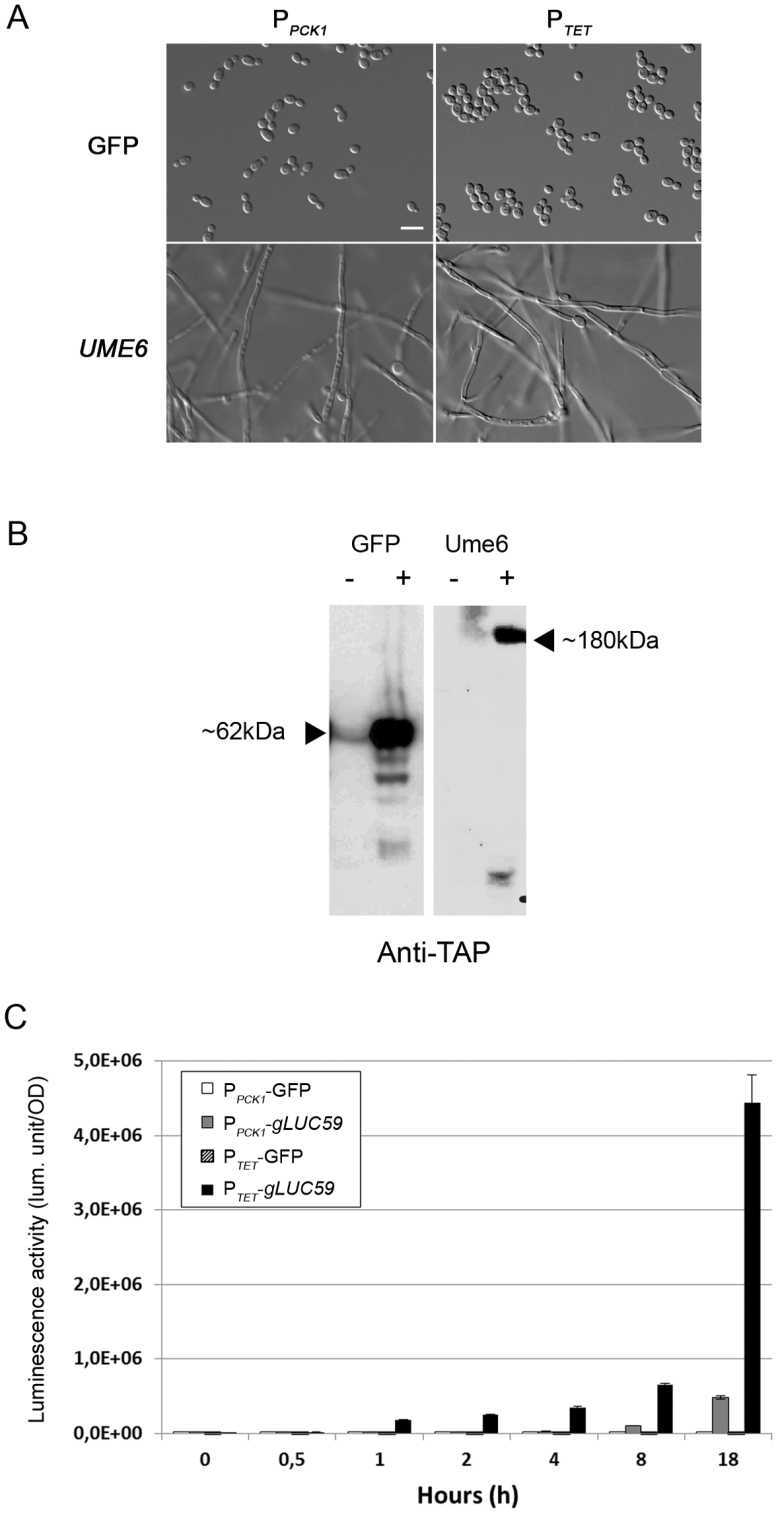
Functionality of the Gateway® OE systems. A. P*_PCK1_*-driven and P*_TET_*-driven OE of *UME6* but not GFP triggers morphogenesis. *C. albicans* strains with integrated CIp10-P*_PCK1_*-GTW-TAPtag or CIp10-P*_TET_*-GTW derivatives harbouring the GFP (CEC2407 or CEC2992, respectively) or *UME6* (CEC1097 or CEC2994, respectively) ORFs were observed microscopically upon growth in gluconeogenic conditions or YPD supplemented with 50 µg.mL**^−^** ^1^ Dox at 30°C for 18 h. Scale bar = 5 µm. **B. Production of TAPtagged proteins.** *C. albicans* strains with integrated CIp10-P*_PCK1_*-GTW-TAPtag derivatives harbouring the GFP (CEC2407) or *UME6* (CEC1097) ORFs were grown in SD (−) or YNB 2% casamino acids (+) for 6 h. Whole cell extracts were separated by SDS-PAGE and probed with a peroxidase-coupled antibody allowing the detection of TAPtagged proteins in gluconeogenic conditions only. Proteins of interest are indicated by an arrow along with their deduced size. **C. Kinetics of expression from the** ***PCK1*** **or** ***TET*** **promoters.** *C. albicans* strains with integrated CIp10-P*_PCK1_*-GTW-TAPtag or CIp10-P*_TET_*-GTW derivatives harbouring the GFP (CEC2407 or CEC2992, respectively) or *gLUC59* (CEC1906 or CEC3083, respectively) ORFs were grown in YNB 2% casamino acids or YPD supplemented with 3 µg.mL**^−^** ^1^ ATc for 18 h at 30°C. Data represent luciferase specific activity detected from the different strains at the indicated time points of growth under inducing conditions. Assays were performed in duplicate and means and SD are shown.

We verified that Gateway®-cloning of ORFs into both plasmids allowed efficient OE of proteins by transferring the GFP [42] and *UME6* ORFs into these vectors. *UME6* was selected as its OE has been shown to trigger hyphal formation [43], [44]. As shown in Fig. 2A, OE of *UME6* resulted in the formation of hypha in conditions that do not normally trigger *C. albicans* morphogenesis. Production of TAP-tagged GFP and Ume6 proteins was also observed in strains harbouring derivatives of CIp10-P*_PCK1_*-GTW-TAPtag and grown under gluconeogenic conditions (Fig. 2B).

We also compared the strengths and expression kinetics of the P*_PCK1_* and P*_TET_* promoters. Strains harbouring a P*_PCK1_-gLUC59* fusion or a P*_TET_-gLUC59* fusion and pNIMX were shifted to gluconeogenic conditions or grown in the presence of 3 µg.mL**^−^** ^1^ anhydrotetracycline (ATc), respectively, and luciferase activity was recorded at different time points following the shift. Results in Fig. 2C showed that an increase in luciferase activity was detectable after 1 h when using P*_TET_* while 4–8 h were needed to see such an increase when using P*_PCK1_*. Moreover, expression levels obtained from P*_TET_* were *ca.* 50 times those achieved from P*_PCK1_* after 18 h of induction. Finally, a 460-fold and 24600-fold induction was observed with P*_PCK1_* and P*_TET_* respectively after 18 h of induction. Thus, these two systems provide versatility in the levels and conditions of OE that can be advantageous when testing the effect of gene OE on a given phenotype.

### Establishment of a Collection of *Candida albicans* OE Strains

**Figure 3:**
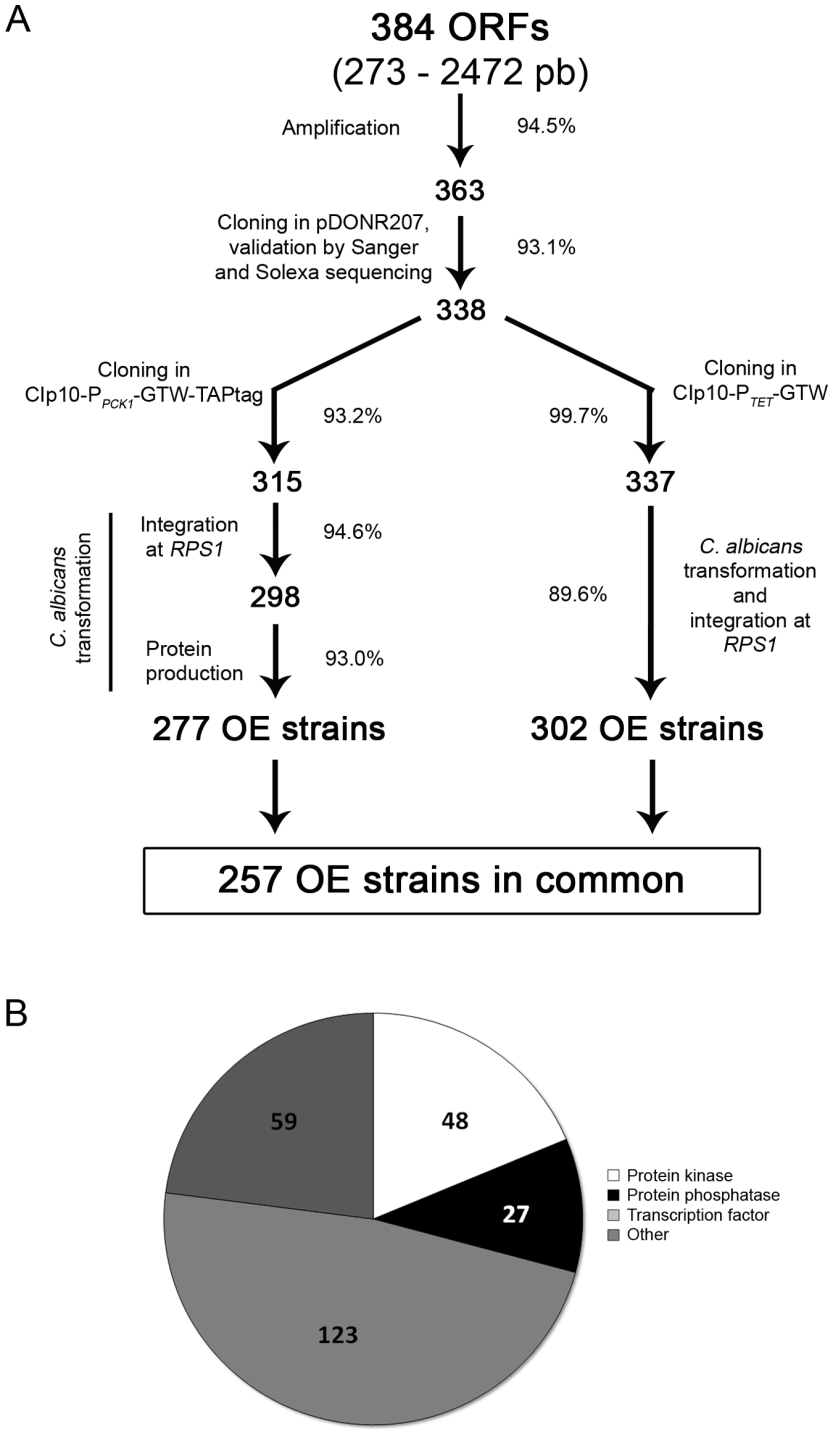
Establishment of two collections of *C. albicans* OE strains. A. Schematic of the OE strain construction pipeline. *C. albicans* ORFs were amplified from their start codon to their penultimate codon and cloned into the pDONR207 vector using Gateway®-mediated recombination. The resulting plasmids were analyzed individually by Sanger sequencing of the ORF-5′ and 3′ ends and in pools by Illumina/Solexa sequencing. Validated ORFs were transferred into CIp10-P*_PCK1_*-GTW-TAPtag or CIp10-P*_TET_*-GTW and the resulting plasmids were introduced at the *RPS1* locus in *C. albicans* strain CEC161 or CEC2907, respectively. Production of a TAPtagged protein of the appropriate size was subsequently tested by Western-blot analysis of protein extracts of the *C. albicans* OE strains grown in YNB +2% casamino acids medium for 18 h in the case of the P*_PCK1_*-driven OE strains. For each step the success rate is indicated along with the number of validated plasmids or strains that have been obtained. **B. Distribution of the 257 overexpressed ORFs overlapping both collections across functional categories.**

Based on these results, we generated two new collections of *C. albicans* OE strains. We focused our study on 384 *C. albicans* ORFs encoding 76 protein kinases (PKs), 36 protein phosphatases (PPs), 179 transcription factors (TFs) and 93 other proteins related to signalling. Corresponding PCR products from the start codon to the penultimate codon were cloned into the pDONR207 donor vector. Following Sanger and Illumina/Solexa sequence validation, a total of 338 (93.1%) derivatives of pDONR207 were obtained (Fig. 3A and Table S1).

ORFs cloned into pDONR207 were subsequently transferred into the CIp10-P*_PCK1_*-GTW-TAPtag and uniquely barcoded CIp10-P*_TET_*-GTW plasmids. A total of 315 CIp10-P*_PCK1_*-GTW-TAPtag derivatives and 337 CIp10-P*_TET_*-GTW derivatives were obtained (Fig. 3A; Table S1). These plasmids were subsequently introduced at the *RPS1* locus in *C. albicans* wild-type strains CEC161 or CEC2907, respectively. Of the resulting 298 strains that harboured a CIp10-P*_PCK1_*-GTW-TAPtag derivative, 277 produced a TAP-tagged protein when grown in gluconeogenic conditions with approx. 15% showing relatively low levels of protein production (Fig. 3 and data not shown). Eventually, 277 *C. albicans* P*_PCK1_*-driven OE strains and 302 *C. albicans* P*_TET_*-driven OE strains were obtained, with 257 genes being represented in both sets of strains (Fig. 3A; Table S1). In summary, our procedure had more than 70% success rate in both cases, reflecting a near 90% success rate at each step. Results presented below focus on the phenotypes associated with the OE of those 257 genes for which P*_PCK1_*-driven and P*_TET_*-driven OE strains were available. These genes encode 48 PKs (44% of all annotated *C. albicans* PKs), 27 PPs (66% of all annotated *C. albicans* PPs), 123 TFs (51% of all annotated *C. albicans* TFs) and 59 other proteins related to signalling (Fig. 3B).

### Screening for Genes Affecting Morphogenesis upon P*_PCK1_*-driven OE

**Figure 4:**
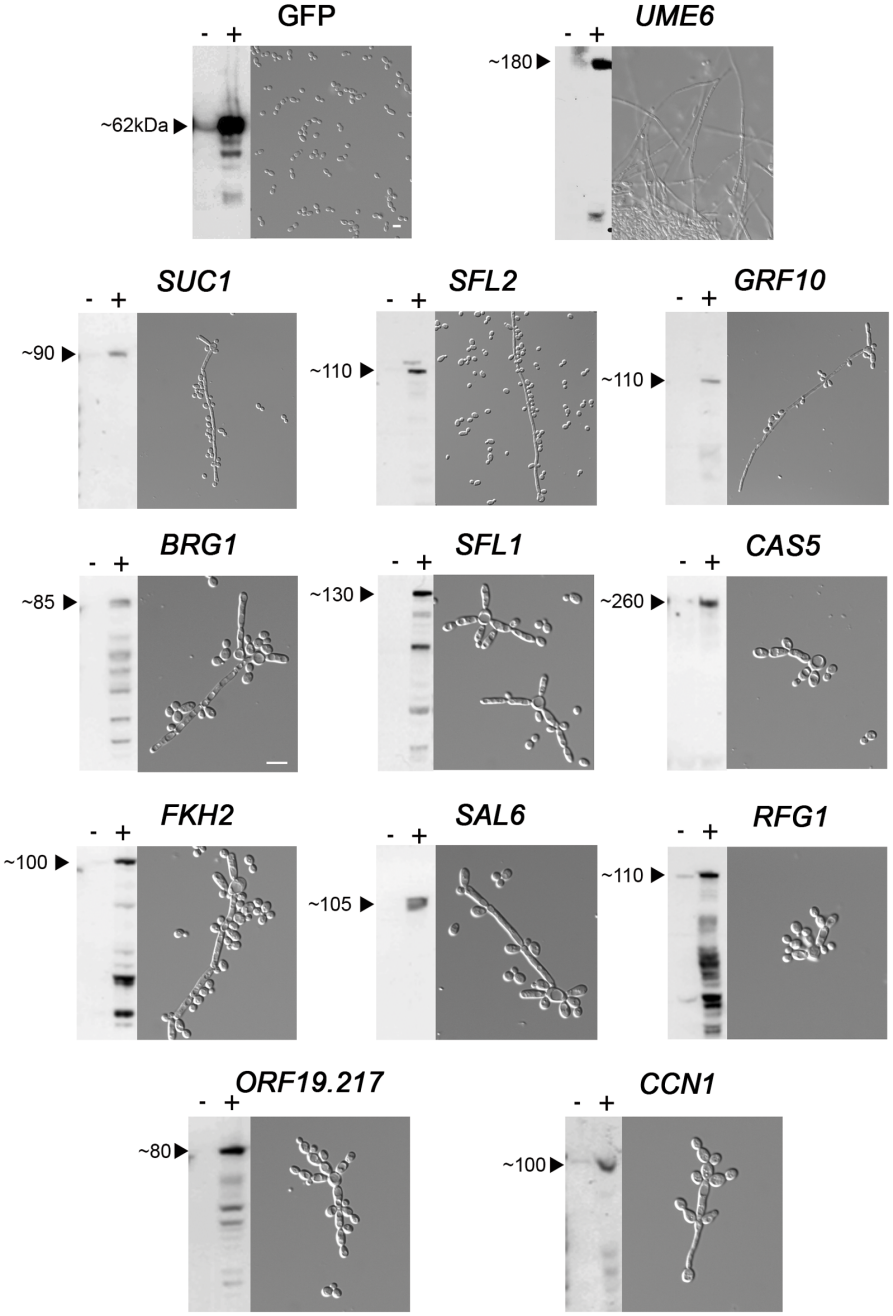
P*_PCK1_*-driven OE of 11 *C. albicans* genes triggers pseudohyphal or hyphal growth. *C. albicans* strains with integrated CIp10-P*_PCK1_*-GTW-TAPtag derivatives harbouring ORFs for the indicated genes were grown in SD (−) or YNB 2% casamino acids (+) for 6 h (Western Blot) or in YNB 2% casamino acids for 18 h (microscopy). Whole cell extracts of uninduced and induced cultures were separated by SDS-PAGE and probed with a peroxidase-coupled antibody allowing the detection of TAPtagged proteins in gluconeogenic conditions only. Proteins of interest are indicated by an arrow along with their deduced size. 18 h induced cultures were observed microscopically and revealed OE-associated pseudo-filamentation or filamentation. Note that the 5 µm scale bar is different for photos of the two upper panels (GFP, *UME6*, *SUC1*, *SFL2*, and *GRF10*) and the rest of the figure.

The ability of *C. albicans* to switch between yeast and hyphal forms is considered a major requirement for virulence and biofilm formation [11], [45]–[48]. Thus, we performed a screen to identify *C. albicans* genes whose P*_PCK1_*-driven OE triggers pseudohyphal or hyphal growth under conditions that normally promote yeast growth. As shown in Fig. 2A and 4, gluconeogenic conditions required for expression from P*_PCK1_* are associated with growth in the yeast form only. Hence, the 257 *C. albicans* P*_PCK1_*-OE strains described above were grown individually in YNB 2% casamino acids at 30°C for 18 h and the cultures were observed microscopically. Eleven strains displayed pseudohyphal or hyphal growth in inducing conditions as shown in Fig. 4. The corresponding genes are listed in Table S2 and included 9 TFs, 1 PP and 1 PK regulatory subunit.

Seven of these genes have been previously associated with morphogenesis including those encoding the Ccn1 G1 cyclin and the Cas5, Fkh2, Rfg1, Sfl1, Sfl2 and Brg1 transcription factors (Table S2). Indeed, inactivation of *CCN1*, *CAS5*, *FKH2*, *SFL2* and *BRG1* results in defects in filamentation [13], [17], [30], [34], [49]–[54]. On the other hand, Sfl1 and Rfg1 have been described as repressors of filamentation in *C. albicans* [10], [13], [55]–[58]. However, the role of *RFG1* is not restricted to this function since its OE triggers pseudohyphal growth [59]. Noticeably, OE of *SFL2* and *BRG1* has been previously shown to trigger hyphal growth [30], [54], [60], [61]. In contrast, several genes identified in this screen were not known for their role in morphogenesis including those encoding the Sal6 phosphatase and the Suc1, Grf10, and Orf19.217 putative transcription factors (Table S2). Interestingly, we have observed that inactivation of *GRF10* and *ORF19.217* did not impair morphogenesis on a variety of hypha-inducing media despite the effect of their OE on morphogenesis (data not shown). These observations are concordant with results published by Homann *et* *al*. [13]. Thus, these results confirmed previous published data obtained with either KO or OE strategies and indicated that our OE approach could reveal genes with novel roles in *C. albicans* pseudohyphal or hyphal differentiation.

### Screening for Genes Affecting Morphogenesis in Liquid Medium upon P*_TET_*-driven OE

**Figure 5:**
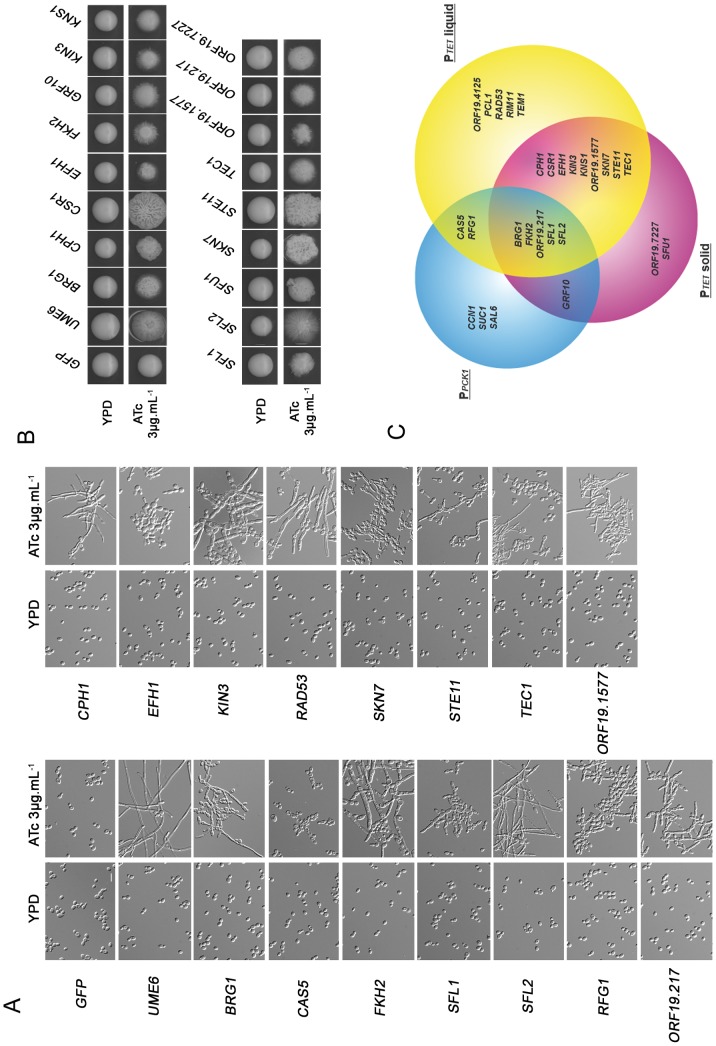
P*_TET_*-driven OE screens confirm results obtained with the P*_PCK1_* promoter and reveal the role of other *C. albicans* genes in morphogenesis. A. P*_TET_*-driven OE of 21 genes promotes pseudo-filamentation or filamentation in liquid media. *C. albicans* strains with integrated CIp10-P*_TET_*-GTW derivatives harbouring ORFs for the indicated genes were grown in YPD or YPD supplemented with 3 µg.mL**^−^** ^1^ ATc for 18 h. Both cultures were observed microscopically and revealed OE-associated pseudofilamentation or filamentation. Genes whose P*_PCK1_*-driven OE results in pseudo-hyphal or hyphal growth are shown on the left panel whereas others genes are placed on the right panel. OE of 15 genes showing the strongest phenotypes are represented, the other 6 are shown in Fig. S2. Scale bar = 5 µm. **B. P** ***_TET_*** **-driven OE of 17 genes promotes filamentation on solid media.** Cultures of *C. albicans* wild-type strains with integrated CIp10-P*_TET_*-GTW derivatives harbouring ORFs for the indicated genes were spotted on YPD or YPD supplemented with 3 µg.mL**^−^** ^1^ ATc and were observed after 5 days of growth at 30°C. C. Overlap between the three morphogenesis screens. This venn diagram was obtained with the online software Gliffy (http://www.gliffy.com) and summarises results obtained in the screens performed with the P*_PCK1_* and P*_TET_* promoters. Circle size is proportional to the number of genes identified.

Next, we performed a similar screen with the P*_TET_*-driven OE collection. Indeed, gene OE from P*_TET_* is highly advantageous since it can be used in any medium supplemented with a tetracycline derivative, while P*_PCK1_*-driven OE is strictly dependent upon gluconeogenic growth conditions. Moreover, we have observed that the level of OE is considerably higher with the P*_TET_* system (Fig. 2C). Thus, the 257 *C. albicans* P*_TET_*-driven OE strains were grown individually in liquid YPD supplemented with 3 µg.mL**^−^** ^1^ ATc at 30°C for 18 h and the cultures were observed microscopically. In these conditions, we observed that P*_TET_*-driven OE of 21 genes induced filamentation or pseudofilamentation (Fig. 5A and Table S2), among which 6 exhibited a weak phenotype (Fig. S2). This gene set included *BRG1*, *SFL2*, *SFL1*, *RFG1*, *CAS5*, *FKH2* and *ORF19.217* already identified in our screen of P*_PCK1_*-driven OE strains. In contrast, we did not observe filamentation upon P*_TET_*-driven OE of *CAS5*, *GRF10*, *SAL6* and *CCN1*. Finally, P*_TET_*-driven OE of 14 additional genes triggered pseudofilamentation and/or filamentation (Fig. 5A, Fig. S2 and Table S2). These included *TEC1*, *EFH1*, *CPH1, PCL1, RAD53, SKN7* and *STE11* whose role in morphogenesis was previously uncovered using KO [17], [18], [30], [62]–[72] and OE mutants [30], [45], [65]–[67]. *TEC1* and *CPH1* are well-characterized regulators of morphogenesis. *TEC1* encodes a TEA/ATTS transcription factor regulating hypha-specific genes as well as biofilm formation and pheromone signalling [11], [29], [64]. *CPH1* encodes a transcription factor required for mating and hyphal growth on solid media and lies in the same Cek1-MAPK pathway than the Ste11 protein [62], [73], [74]. *PCL1* encodes a cyclin homolog whose expression is induced upon filamentous growth [56]. It was also recently shown to be required for agar invasion at elevated temperature [72]. The Rad53 protein kinase is involved in DNA-replication and DNA-damage checkpoint pathways and its deletion is known to nearly completely abolish filamentous growth caused by genotoxic stresses [71]. *SKN7* is predicted to encode a response regulator protein in a phosphorelay signal transduction pathway. Its deletion leads to a morphogenesis defect [68] but it has been mostly associated to a role in the response of *C. albicans* to oxidative stress and osmoregulation [13], [68]. Among the four remaining genes, *RIM11* and *KIN3* encode two previously characterized protein kinases that had not been associated with morphogenesis yet. Of notable interest, our screen uncovered two uncharacterised genes, *ORF19.1577* and *ORF19.4125,* whose OE triggered filamentation. Heterozygous or homozygous mutants for these genes have been obtained [17], [18] but have not been associated to any relevant phenotype except for the heterozygous mutant *ORF19.4125*Δ that presents a reduced ability to invade agar compared to a wild-type strain [18].

### Screening for Genes Affecting Morphogenesis in Solid Medium upon P*_TET_*-driven OE

We additionally performed a screen of our P*_TET_*-driven OE strain collection on YPD solid medium supplemented with 3 µg.mL**^−^** ^1^ATc and identified 17 genes whose OE triggered filamentation (Fig. 5B). Noticeably, three of these genes had not been identified in the screen performed in liquid conditions, namely *SFU1*, *GRF10* and *ORF19.7227*. Moreover, the phenotype associated to the OE of *CSR1* and *KNS1* was relatively weak (Fig. S2). *CSR1* (or *ZAP1*) encodes a zinc-finger TF involved in zinc homeostasis and in regulation of biofilm matrix production [33], [75]. It has been shown that deletion of *CSR1/ZAP1* affects filamentous growth [10], [13], [17], [33], [76]. Similarly, a homozygous transposon insertion in *ORF19.7227* that encodes a putative protein phosphatase inhibitor (PPI) decreases colony wrinkling but does not block true hyphal growth in liquid media [16], consistent with our observations. In contrast, the putative Ser/Thr PK Kns1 and the TF Sfu1 had not been previously associated to a function in filamentous growth. Indeed, *KNS1* remains uncharacterized and *SFU1* encodes a transcriptional repressor of iron-responsive genes [77]. Finally, we also noted that five of the 18 genes identified in the screen in liquid medium were not recovered in the screen on solid medium (*PCL1, RFG1, CAS5, RIM11, ORF19.4125*; Fig. 5A and B).

Taken together, the three screens performed using our P*_TET_*-driven and P*_PCK1_*-driven OE strain collections showed overlaps but specificities in the sets of genes that were identified based on the impact of their OE on morphogenesis (Fig. 5C), reemphasizing the interest of using versatile OE vectors. Moreover, all three screens revealed genes with previously unknown roles in morphogenesis, confirming the potential of an OE approach for *C. albicans* functional genomics.

### Impact of P*_TET_*-driven OE on *C. albicans* Growth Rate

In the yeast *S. cerevisiae*, OE of up to 15% of the gene repertoire results in growth defects at the colony level. This is often due to pathway activation and can be used to identify targets of genes whose OE is toxic [23], [27]. Therefore, we assessed to what extent our P*_TET_*-driven OE system could trigger changes in *C. albicans* growth rate.

**Figure 6:**
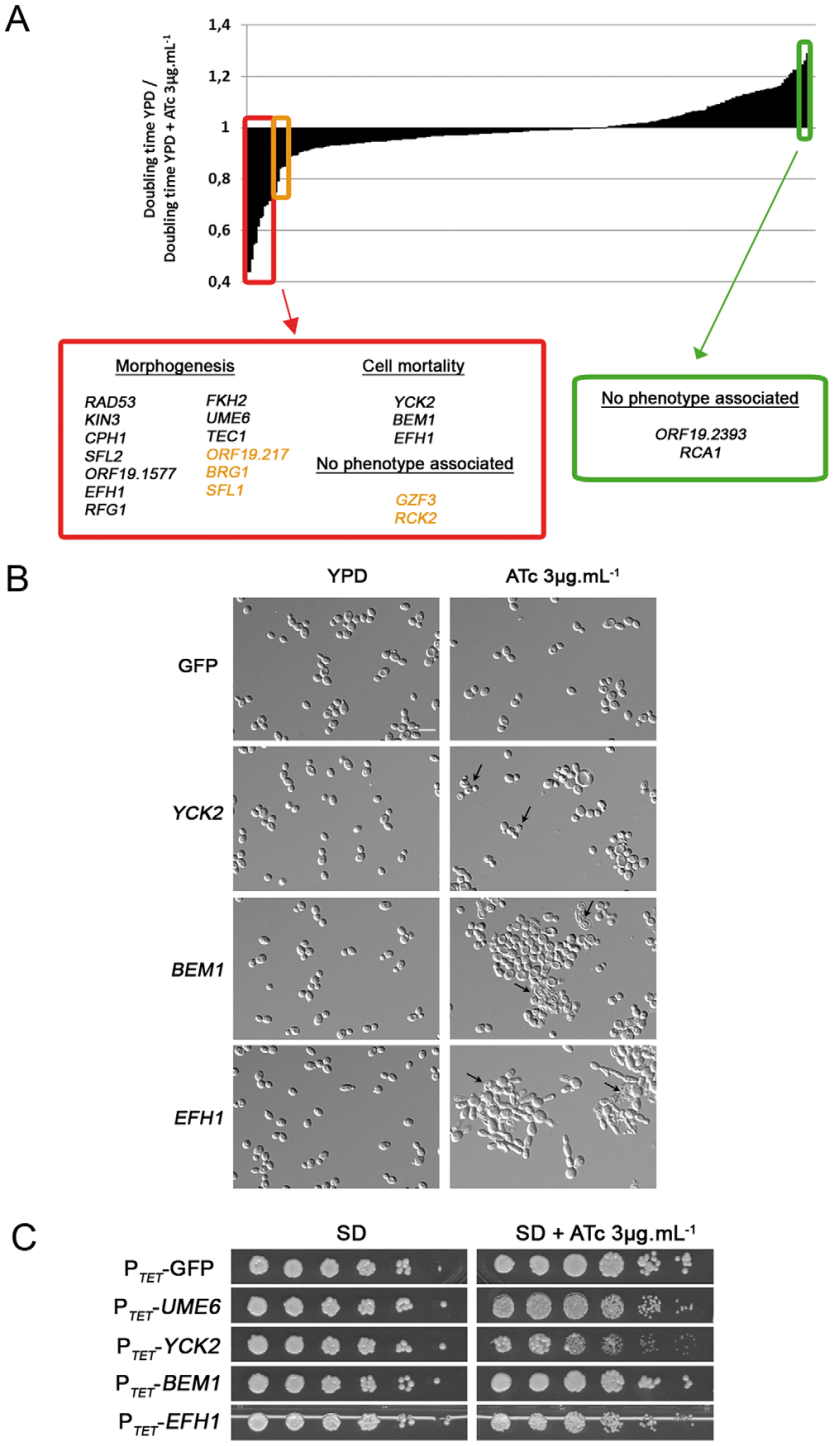
P*_TET_*-driven OE of 20 genes results in decreased or increased growth rate. A. Impact of OE on the growth rates of 257 OE strains. Genes whose OE decreases or increases growth rate (≥2 fold and >2 fold, respectively) upon growth in liquid medium are listed in red or green boxes respectively. Genes are classified based on their OE phenotype from the more affected to the less affected. Genes in orange correspond to those whose OE decreased growth with a fold equal to 2. Data are mean and SD of 3 experiments. **B. P** ***_TET_*** **-driven OE of** ***YCK2*** **,** ***BEM1*** **and** ***EFH1*** **results in cell lysis.** *C. albicans* strains with integrated CIp10-P*_TET_*-GTW derivatives harbouring the *GFP*, *YCK2*, *BEM1* or *EFH1* ORFs were grown in YPD or YPD supplemented with 3 µg.mL**^−^** ^1^ ATc for 18 h and observed microscopically. Cell lysis is indicated by arrows. Scale bar = 5 µm. **C. P** ***_TET_*** **-driven OE of** ***YCK2*** **and** ***EFH1*** **reduces growth rate on solid medium.** Serial dilutions of cultures of *C. albicans* strains with integrated CIp10-P*_TET_*-GTW derivatives harbouring the *GFP*, *UME6*, *YCK2*, *BEM1* or *EFH1* ORFs were spotted on SD or SD supplemented with 3 µg.mL**^−^** ^1^ ATc and observed after 3 days of growth at 30°C.

The 257 P*_TET_*-driven OE strains were surveyed during a growth kinetic in 96-well plates in the presence or absence of ATc. For each strain, a ratio equalliing to the doubling time monitored in non-inducing conditions (YPD) divided by the doubling time observed under inducing conditions (YPD supplemented with 3 µg.mL**^−^** ^1^ ATc) was calculated. This identified 17 genes whose OE decreased *C. albicans* growth rate (≥2 fold) and 2 genes whose OE increased *C. albicans* growth rate (>2 fold) (Fig. 6A). The latter two genes encode the bZIP domain-containing protein of the ATF/CREB family Rca1 and a putative TF of unknown function, Orf19.2393 (Fig. 6A). We did not observe other phenotypes associated with the OE of these genes in a wild-type strain (data not shown). The *rca1*ΔΔ mutant is viable but slow-growing and displays increased invasive growth [13], [17], consistent with our observations. This gene clearly plays important roles in *C. albicans* biology since it controls both the susceptibility to different antifungals [20] and carbonic anhydrase expression via the cAMP/PKA/Efg1 signalling pathway [78].

A majority of the genes whose OE resulted in decreased growth rate (13/17 genes or 76.4%) were among those whose OE triggered filamentation (Fig. 5A, 5B and 6A). Indeed, filamentation results in optical density readings that are not correlated with the actual growth rates. Nevertheless, we observed cell death when the *BEM1*, *YCK2* or *EFH1* genes were overexpressed (Fig. 6B). Decreased growth was also observed when strains overexpressing *YCK2* or *EFH1* were grown on solid medium (Fig. 6C). *BEM1* has previously been shown to be essential in *C. albicans* [79], [80]. Despite the requirement of this gene for pseudohyphal and hyphal growth in *S. cerevisiae* and *Yarrowia lipolytica*, respectively [81], [82], the role of Bem1 in *C. albicans* morphogenesis remains unclear [79], [80]. None of the two other genes (*YCK2* and *EFH1*) were previously associated with cell growth. In particular, *YCK2* encodes a plasma membrane protein similar to the highly conserved serine/threonine casein kinase 1 (CK1) of *S. cerevisiae* and plays role in damaging oral epithelial cells and hyphal branching [83]. Thus, our results revealed genes involved in *C. albicans* fitness, including genes not previously described for such a role. However, if one excludes genes whose OE triggers morphogenesis, these genes represent a minor fraction of those we have tested. This is despite the fact that many of them have regulatory functions and suggests that *C. albicans* might be more robust than *S. cerevisiae* to the deleterious effects of gene OE and/or that our OE system is not sufficient to reveal such phenotypes.

### Conclusions

In this study, we have reported the development of two collections of OE strains and the potential of OE screens in uncovering novel components of regulatory pathways in *C. albicans*. To date, OE screens have been rarely used for the identification of genes conferring specific phenotypes in *C. albicans*. Fu *et al*. [28] have established a collection of 26 *C. albicans* OE strains whereby genes encoding GPI-anchored proteins are overexpressed from a tetracycline-repressible promoter [84]. Sahni *et al*. [29] have constructed a collection of 107 *C. albicans* OE strains whereby genes encoding transcription factors are overexpressed from a tetracycline-inducible promoter [39]. These collections were developed using promoter replacement at the targeted gene through a split-marker strategy [28] or allelic exchange between an *ADH1* allele and an OE plasmid obtained by restriction enzyme mediated cloning [39]. Therefore, these resources lack some of the versatility and reusability that is associated with the partial *C. albicans* ORFeome and collections of OE plasmids developed here. Indeed, our strategy was based on the highly efficient Gateway® recombinational cloning methodology [35], [85] that provided the possibility to shuttle ORFs between plasmids allowing gluconeogenesis- or tetracycline-inducible expression and production of tagged or untagged proteins. Moreover, because our OE plasmids used an integrative vector that is targeted highly efficiently to the *C. albicans RPS1* locus, development of collections of OE strains in various genetic backgrounds is rather straightforward (AN, SBB and CE, unpublished data). Hence, our work has laid the ground for the establishment of a *C. albicans* ORFeome and a genome-wide collection of *C. albicans* OE strains, a collaborative project that is ongoing in our laboratory and that of C. Munro (University of Aberdeen; [86]).

Several of the genes that we have identified in our morphogenesis alteration screen were already known for their role in filamentation such as *BRG1*, *CPH1, SFL2* and *TEC1*, thus validating our screen (Table S2). However, by comparison with previous studies, we noticed that OE of three genes present in our collection, namely *RAS1, GPR1* and *TPK2*, and known to trigger morphogenesis upon OE [87]–[89] did not result in pseudohyphal or hyphal growth here. Indeed, we observed that amplification was associated with one mismatch every 1286 bp on average (see Materials and Methods for details) and certain genes harboured non-synonymous mutations (Table S1) that might have impacted their function. Moreover, we noticed some differences in the OE phenotypes according to the promoters we have used. This may be explained by 1) the difference of expression between the *PCK1* and *TET* promoters, 2) the variable level of expression between clones of the same transformation due to tandem insertions and 3) the fact that some P*_PCK1_*-driven overexpressed proteins may not be fully functional because of the occurrence of the TAPtag at their carboxy-terminus. In conclusion, the *TET* promoter appears the most suitable system for large-scale OE screens for different reasons: 1) its induction is simpler and easier to control; 2) it provides a higher level of induction compared to the *PCK1* promoter system; 3) it has been shown to function in animal models of *C. albicans* infections.

Among the 257 genes that we have tested, at least 137 have also been characterized by gene deletion and 34 (24.8%) were shown to be involved to some extent in morphogenesis [13], [17]. It is noteworthy that for a subset of the genes that we have identified based on the impact of their OE on morphogenesis the corresponding KO mutations did not impair morphogenesis (such as *GRF10* and *ORF19.217*; [13] and data not shown). This highlights the complementarity between KO and OE screens and reinforces the potential of the latter in exploring regulatory networks [90].

In summary, this study provides an example of the potential of OE approaches in the investigation of *C. albicans* biology. Taken together, our results highlight the multiplicity of possible applications of our strategy such as a variety of phenotypic screens in *C. albicans*. In the short term, the development of diverse destination vectors and recipient strains will help to rapidly determine the function of a specific gene or to identify its partners, *eg* using suppressor screens or two-hybrid screens [26]. Moreover, the presence of barcodes in our P*_TET_*-driven OE strain collection will enable experiments in pools that will greatly facilitate large-scale experiments. Overall, the strain collections generated during this study and available through the Fungal Genetics Stock Center [91], as well as the OE strategy we developed, will allow the generation of a large number of relevant data for the whole *Candida* community.

## Materials and Methods

### Strains and Media

All *C. albicans* strains used in this study are listed in Table 1. Strains were grown at 30°C in YPD medium (1% yeast extract, 2% peptone, 2% glucose) or SD minimal medium [0.67% yeast nitrogen base (YNB; Difco) with 0.4 or 2% glucose] supplemented if necessary with arginine, histidine and uridine, at 20 mg.L**^−^** ^1^ and 2% agar for solid media. OE from P*_PCK1_* was triggered in YNB plus 2% casamino acids liquid cultures at 30°C whereas OE from P*_TET_* was induced by the addition of 50 µg.mL**^−^** ^1^ doxycycline (Dox - Fluka) or 3 µg.mL**^−^** ^1^ anhydrotetracycline (ATc - Fisher Bioblock Scientific) in YPD at 30°C. ATc was preferred over Dox as this semi-synthetic tetracycline derivative has been described for its lower toxicity and its higher efficiency in the binding of the TetR repressor protein [92]. Furthermore, we have observed that 2 µg.mL**^−^** ^1^ ATc reproduced the effect of 50 µg.mL**^−^** ^1^ Dox, either on solid or in liquid medium and this concentration was not deleterious for growth or morphogenesis of *C. albicans* (Fig. S1 and data not shown). Dox- and ATc-containing cultures were maintained in the dark as these compounds are light sensitive.

**Table 1:** Strains used in this study.

Strain name	Genotype	References
BWP17	*ura3*Δ*::λimm434/ura3*Δ*::λimm434 his1*Δ*::λimm434/his1*Δ*::λimm434 arg4*Δ*::λimm434/arg4*Δ*::λimm434 iro1*Δ*::λimm434/* *iro1*Δ*::λimm433*	[101]
CEC161	*ura3*Δ*::λimm434/ura3*Δ*::λimm434 his1*Δ*::hisG/HIS1 arg4*Δ*::hisG/ARG4*	[102]
CEC955	*ura3*Δ*::λimm434/ura3*Δ*::λimm434 his1*Δ*::hisG/HIS1 arg4*Δ*::hisG/ARG4 ADH1/adh1::ADH1p-cartTA::SAT1::P_TET_-caGFP*	This study
CEC988	*ura3*Δ*::λimm434/ura3*Δ*::λimm434 ARG4/arg4*Δ*::hisG HIS1/his1*Δ*::hisG RPS1/RPS1::CIp10-P_ACT1_-gLUC59*	This study
CEC1097	*ura3*Δ*::λimm434/ura3*Δ*::λimm434 arg4*Δ*::hisG/ARG4 his1*Δ*::hisG/HIS1 RPS1/RPS1::CIp10-P_PCK1_-UME6-TAPtag*	This study
CEC1906	*ura3*Δ*::λimm434/ura3*Δ*::λimm434 arg4*Δ*::hisG/ARG4 his1*Δ*::hisG/HIS1 RPS1/RPS1::CIp10-P_PCK1_-gLUC59-TAPtag*	This study
CEC1909	*ura3*Δ*::λimm434/ura3*Δ*::λimm434 his1*Δ*::hisG/HIS1 arg4*Δ*::hisG/ARG4 ADH1/adh1::ADH1p-cartTA::SAT1::P_TET_-caGFP RPS1/* *RPS1::P_TET_ -gLUC59*	This study
CEC2175	*ura3*Δ*::λimm434/ura3*Δ*::λimm434 his1*Δ*::hisG/HIS1 arg4*Δ*::hisG/ARG4 ADH1/adh1:: P_ADH1_-cartTA::SAT1*	This study
CEC2249	*ura3*Δ*::λimm434/ura3*Δ*::λimm434 his1*Δ*::hisG/HIS1 arg4*Δ*::hisG/ARG4 ADH1/adh1::ADH1p-cartTA::SAT1 RPS1/RPS1:: P_TET_ -gLUC59*	This study
CEC2407	*ura3*Δ*::λimm434/ura3*Δ*::λimm434 his1*Δ*::hisG/HIS1 arg4*Δ*::hisG/ARG4 RPS1/RPS1::CIp10-P_PCK1_-GFP-TAPtag*	This study
CEC2907 - CEC2908	*ura3*Δ*::λimm434/ura3*Δ*::λimm434 his1*Δ*::hisG/HIS1 arg4*Δ*::hisG/ARG4 ADH1/adh1::P_TDH3_-carTA::SAT1*	This study
CEC2992	*ura3*Δ*::λimm434/ura3*Δ*::λimm434 his1*Δ*::hisG/HIS1 arg4*Δ*::hisG/ARG4 ADH1/adh1::P_TDH3_-carTA::SAT1 RPS1/RPS1::CIp10-P_TET_-GFP*	This study
CEC2994	*ura3*Δ*::λimm434/ura3*Δ*::λimm434 his1*Δ*::hisG/HIS1 arg4*Δ*::hisG/ARG4 ADH1/adh1::P_TDH3_-carTA::SAT1 RPS1/RPS1::CIp10-P_TET_-UME6*	This study
CEC3083	*ura3*Δ*::λimm434/ura3*Δ*::λimm434 his1*Δ*::hisG/HIS1 arg4*Δ*::hisG/ARG4 ADH1/adh1:: P_ADH1_-cartTA::SAT1 RPS1/RPS1:: P_TET_ -gLUC59*	This study

Plasmids harbouring a Gateway® cassette were propagated in *Escherichia coli* strain TOP10 *ccdB^R^* (Invitrogen). Other plasmids were propagated in *E. coli* strain DH5α [93]. *E. coli* strains were grown in LB medium. Antibiotics were used at the following concentrations: ticarcillin, 50 µg.mL**^−^** ^1^; gentamycin, 10 µg.mL**^−^** ^1^; chloramphenicol, 15 µg.mL**^−^** ^1^.

### Construction of a *C. albicans* Partial ORFeome

The detailed method for the cloning of *C. albicans* ORFs in the pDONR207 vector has been described [94]. Briefly, for each of the selected ORF, a forward primer including the *attB1* site and the first 10 codons of the ORF and a reverse primer including the *attB2* site and the last ten codons of the ORF were designed and synthesized at Pasteur-Genopole-Ile-de-France oligonucleotide synthesis platform (Table S1). ORFs were amplified from genomic DNA of *C. albicans* strain SC5314 [95] using Eppendorf Triple Master Taq polymerase and 30 cycles of amplification with elongation time varying from 1 to 3 min. according to the ORF size. The resulting PCR products were checked by agarose gel electrophoresis, ethanol precipitated and, following resuspension in Tris-EDTA (TE), mixed with the donor plasmid pDONR207 (Invitrogen), and subjected to a recombination reaction with Invitrogen Gateway® BP Clonase™. The recombination mixes were transformed into *E. coli* strain DH5αand one transformant per ORF was selected for further study. Plasmids were prepared using the Millipore™ MultiScreen™ HTS 96-well Filtration System and Millipore™ MultiScreen™ PLASMID. The cloned ORFs were sequenced from the 5′- and 3′-ends using Sanger sequencing. Moreover, a pool of the 347 plasmids was subjected to Illumina/Solexa sequencing in order to obtain full length sequencing of the ORFs. Sequencing reads were aligned to the ORF sequences available from the *Candida* Genome Database [96] using CLC Genomics Workbench version 4. Polymorphisms were compared to a database of SNPs obtained following Illumina/Solexa sequencing of *C. albicans* strain SC5314 (Diogo *et al*., manuscript in preparation). All plasmids with mutations causing a non-sense mutation or a frame-shift were excluded. Among the remaining 312 plasmids, we detected 494 synonymous and 405 non-synonymous mismatches. Among these, 362 synonymous and 209 non-synonymous mismatches had also been identified by Solexa/Illumina sequencing of strain SC5314 suggesting that they correspond to genuine SNPs. This indicated that our cloning procedure was responsible for 132 synonymous and 196 non-synonymous mutations in 422 kb insert sequences corresponding to one mutation at every 1286 bp. Information on these mutations is available in Table S1.

### Construction of Gateway®-compatible *C. albicans* OE Vectors

The sequences of oligonucleotides used for cloning purposes are listed in Table S3. Two Gateway®-compatible vectors for conditional OE in *C. albicans* were constructed. First, oligonucleotides Nco-5′Sce and Nco-3′Sce were annealed and inserted into the NcoI site of the *C. albicans* CIp10 integrative vector [36]. This vector designated CIp10S is bearing the 18 bp *I-Sce*I site that is not found in the *C. albicans* genome. Then, the *C. albicans PCK1* promoter region (P*_PCK1_*) was amplified from *C. albicans* strain SC5314 genomic DNA using primers PRPKC1PR and TAPFUR. The TAPtag coding region was amplified using oligonucleotides TERPVUII and TAPFUF and plasmid pFA-TAP-URA3, a derivative of pFA-GFP-URA3 [97] where the *Pst*I/*Asc*I fragment harbouring the GFP coding region has been replaced by a *Pst*I/*Asc*I fragment carrying the TAPtag coding region amplified from plasmid pBS1479 [38] using oligonucleotides Tap1-PstI and Tap2-AscI that allow the addition of a (Gly-Ala)_3_ coding linker 5′ of the TAPtag coding sequence. Both PCR products were mixed and a fusion product was amplified using primers TERPVUII and PRPKC1PR and cloned into the TOPO-TA cloning vector (Invitrogen). The *Kpn*I/*Pvu*II P*_PCK1_*-TAPtag cassette was excised from the resulting plasmid and cloned into *Kpn*I/*Eco*RV-digested CIp10S, yielding CIp10-P*_PCK1_*-TAPtag. The Gateway® RfB cassette was excised from pBS-RfB using *Eco*RV and cloned into *Eco*RV-digested CIp10-P*_PCK1_*-TAPtag, yielding CIp10-P*_PCK1_*-GTW-TAPtag. In order to construct the CIp10-P*_TET_*-GTW plasmid, a tetracycline-inducible promotor (P*_TET_*) was amplified from plasmid pTET25 [39] using oligonucleotides TETKpn and TetATGE5, and cloned into *Kpn*I/*Eco*RV-digested CIp10-P*_PCK1_*-GTW-TAPtag, yielding CIp10S-P*_TET_*-TAPtag. This vector was amplified using Vect32 and Vect33 and the PCR product was digested with *Eco*RV and self-ligated yielding plasmid CIp10S-P*_TET_* that has three stop codons downstream of the *Eco*RV site. The *Eco*RV-digested Gateway® RfB cassette was cloned into *Eco*RV-digested CIp10S-P*_TET_*, yielding CIp10S-P*_TET_*-GTW. Subsequently, a derivative of CIp10S-P*_TET_*-GTW was constructed by *Stu*I digestion and ligation of the annealed Vect30 and Vect31 oligonucleotides. This vector was designated CIp10-P*_TET_*-GTW. In this vector, the *I-Sce*I site is closer to the *RPS1* sequences that are used for integration at the *C. albicans RPS1* locus than in the CIp10-P*_PCK1_*-GTW-TAPtag. Hence transformation efficiency and integration at the *RPS1* locus are higher when using *I-Sce*I-digested CIp10-P*_TET_*-GTW derivatives as compared to *I-Sce*I-digested CIp10-P*_PCK1_*-GTW-TAPtag derivatives (data not shown). Yet, the use of *Stu*I digestion to target derivatives of these plasmids at the *RPS1* locus is still preferred.

A collection of CIp10-P*_TET_*-GTW derivatives was generated by the incorporation of specific molecular barcodes. We used the set of molecular barcodes previously designed for the construction of the *S. cerevisiae* deletion collections [98]. Briefly, these barcodes consist of a specific 20 bp sequence flanked by universal primer sequences (U1 and U2 or D1 and D2). These barcodes were amplified by PCR using genomic DNA prepared from a pool of the *S. cerevisiae* heterozygous deletion collection and primers Sac-U1 and Sac-U2 or Sac-D1 and Sac-D2. The resulting PCR products were digested with *Sac*II and ligated into *Sac*II-digested and dephosphorylated CIp10-P*_TET_*-GTW. Individual clones were recovered after *E. coli* TOP10 *ccdB^R^* transformation and the cloned barcodes were sequenced. Only plasmids with a tag showing a unique ID in the TAG4 yeast barcode array and without mismatch in the common primers U1-U2 or D1-D2 were kept. In total, 936 barcoded derivatives of CIp10-P*_TET_*-GTW were obtained.

### Construction of *C. albicans* OE Strains

Detailed methods for the transfer of *C. albicans* ORFs from pDONR207 into the CIp10-P*_PCK1_*-GTW-TAPtag or barcoded CIp10-P*_TET_*-GTW plasmids as well as the integration of the resulting expression plasmids at the *RPS1* locus have been described [94]. Briefly, an aliquot of each derivative of pDONR207 was mixed with 50 ng of one of the destination plasmids and subjected to a recombination reaction with Invitrogen Gateway® LR Clonase™. The recombination mixes were transformed into *E. coli* strain DH5αand one transformant was used for plasmid preparation as described above. EcoRV digestion was used to verify the cloning of the appropriate ORF. The expression plasmids bearing P*_PCK1_* were digested by StuI (or I-SceI if necessary) and transformed into *C. albicans* strain CEC161 according to Walther and Wendland [99]. Transformants were selected for prototrophy and verified by PCR using primers CIpUL and CIpUR that yield a 1 kb product if integration of the OE plasmid has occurred at the *RPS1* locus. Alternatively, the expression plasmids bearing P*_TET_* were transformed into *C. albicans* strain CEC2907 following *Stu*I or *I-Sce*I linearization. CEC2907 is a derivative strain of CEC161 transformed with pNIMX (Fig. 1B). pNIMX is a derivative of pNIM1 [39] that was modified in two steps. First pNIM1 (Fig. 1B.a) was digested by *Nco*I and *Bgl*II, treated to create blunt ends and self-ligated to reconstitute an *Nco*I site, yielding pNIM1ΔP*_TET_*-GFP (Fig. 1B.b). Next, the *ADH1* promoter (P*_ADH1_*) was replaced by the *TDH3* promoter (P*_TDH3_*) as follows: a region of *cartTA* (containing the start codon) was excised from pNIM1ΔP*_TET_*-GFP using *Acl*I and subcloned into *Cla*I-digested BLUESCRIPT-SK(-)-P*_TDH3_*. The resulting plasmid was linearized with *Xho*I, treated to create blunt ends and digested by *Xba*I. The *Xho*I(blunt)-*Xba*I fragment containing the P*_TDH3_*-*cartTA* fusion was then subcloned in pNIM1ΔP*_TET_*-GFP linearized with *Bam*HI, treated to created blunt ends and digested by *Xba*I, yielding pNIMX (Fig. 1B.c), in which P*_TDH3_* is inserted upstream of *cartTA*. Integration of pNIMX digested with *Kpn*I and *Sac*II at the *ADH1* locus in strains CEC2907 was verified by PCR using primers NIM1_verif and ADH1_verif.

### Analysis of TAPtagged Proteins by Western Blotting

A 20 mL culture in SD or YNB 2% casamino acids was inoculated at OD_600_ = 0.05 with a freshly grown colony. 10 ODs of exponentially growing cells were collected by centrifugation after 4–6 h of growth at 30°C, resuspended in lysis buffer (0.1 M NaOH, 0.5M EDTA, 2% SDS, 2% β-mercaptoethanol) and incubated 10 min at 90°C [100]. The lysate was neutralized with 5 µL 4M acetic acid, incubated 10 min at 90°C and 50 µL Loading buffer (0.25 M Tris-HCl pH 6.8, 50% glycerol, 0.05% bromophenol blue) were added. Proteins were separated on an Invitrogen 10% NuPage gel, transferred onto nitrocellulose and TAPtagged proteins were detected using peroxidase-coupled anti-peroxidase antibodies (Sigma) and an ECL kit (GE Healthcare).

### Luciferase Assays

100 mL of YNB 2% casamino acids or YPD supplemented with 3 µg.mL**^−^** ^1^ ATc were inoculated with a freshly grown colony on SD 2% glucose resuspended in dH_2_O (in the case of P*_PCK1_*-driven OE strains) or an overnight culture in YPD at 30°C (in the case of P*_TET_*-driven OE strains). At each time point, a volume equivalent to 20 OD was centrifuged (2–5 min at 3500 rpm) and resuspended in 200 µL of R-luc buffer (NaCl 0.5 M, Na_2_HPO_4_ 0.1 M pH 6.7, EDTA 1 mM). For luciferase assays, 100 µL of cells were mixed with 20 µL of 2 µM coelenterazine before luminescence (integration time: 1000 ms) and absorbance (wavelength: 610 nm) were measured using a microplate reader (TECAN Infinite 200). The final luminescence value is obtained by the following formula: Luminescence unit/Absorbance.

### Spotting Assays

Strains in the P*_TET_*-driven OE collection were grown in 96-well plate in YPD (30 h; 30°C) and spotted on YPD plates supplemented or not with 3 µg.mL**^−^** ^1^ ATc using the RoTor robot (Singer Instrument). Alternatively, 5-fold serial dilutions of 3 mL overnight cultures in SD at 30°C were spotted on SD plates supplemented or not with 3 µg.mL**^−^** ^1^ ATc. In both cases, plates were grown at 30°C for 2–5 days and scanned with Epson perfection 4490. Spotting assays were performed in triplicate.

### Growth Kinetics

Strains in the P*_TET_*-driven OE collection were grown in 96-well plates in YPD (30 h; 30°C) and inoculated at a final OD_600_ = 0.1 in 100 µL YPD supplemented or not with 3 µg.mL**^−^** ^1^ ATc. Growth at 30°C was monitored every 20 minutes using a microplate reader (TECAN Sunrise). Doubling-time (DT) was calculated by dividing by 2 the time between OD_600_ = 0.15 and OD_600_ = 0.6. Growth curves were performed in triplicate.

### Microscopy and Image Analysis

Cells were observed with a Leica DM RXA microscope (Leica Microsystems). Images were captured with a Hamamatsu ORCA II-ER cooled CCD camera, using the Openlab software version 3.5.1 (Improvision Inc.), and then processed with Adobe Photoshop 10.0 software.

## Supporting Information

Figure S1 **Comparison of doxycycline (Dox) and anhydrotetracycline (ATc). A. 50 µg.mL^−1^ Dox or 2 µg.mL^−1^ ATc induce P** ***_TET_*** **to a similar extent.** *C. albicans* strains with integrated CIp10-P*_TET_*-GTW derivatives harbouring the GFP or *gLUC59* ORFs (CEC2992 or CEC3083, respectively) were grown in YPD supplemented with 50 µg. mL**^−^** ^1^ Dox or 2 µg.mL**^−^** ^1^ ATc for 18 h at 30°C. Data represent luciferase specific activity detected from the different strains at 0 and 18 h of growth under inducing conditions. Assays were performed in duplicate and means and SD are shown. **B. Effects on morphogenesis are similar between 50 µg.mL^−1^ Dox and 2 µg.mL^−1^ ATc.** *C. albicans* strain SC5314 and a strain overexpressing *UME6* (CEC2994) were grown in YPD medium and spotted on YPD medium supplemented or not with tetracycline analog (50 µg.mL**^−^** ^1^ Dox or 2 µg.mL**^−^** ^1^ ATc). Pictures were taken after 5 days of growth at 30°C. **C. ATc shows lower inhibition of** ***C. albicans*** **hyphal growth than Dox.** *C. albicans* strain SC5314 was grown in YPD liquid medium supplemented or not with different concentrations of Dox or ATc for 18 h at 30°C and observed microscopically. Scale bar = 5 µm.
(TIF)

Figure S2 **P** ***_TET_*** **-driven OE of 6 genes leads to a weak but significant phenotype in liquid media.** *C. albicans* strains with integrated CIp10-P*_TET_*-GTW derivatives harbouring ORFs for the indicated genes were grown in YPD or YPD supplemented with 3 µg.mL**^−^** ^1^ ATc for 18 h. Both cultures were observed microscopically and revealed OE-associated pseudofilamentation or filamentation (germ tubes essentially). Scale bar = 5 µm.
(TIF)

Table S1 **Summary of the collections provided in this study.**
(XLSX)

Table S2 ***Candida albicans*** **genes whose P** ***_PCK1_*** **-driven or P** ***_TET_*** **-driven OE triggers pseudohyphal or hyphal growth.**
(DOCX)

Table S3 **Oligonucleotides used in this study.**
(DOCX)
