# Supplementary figures and images for: A Versatile Overexpression Strategy in the Pathogenic Yeast Candida albicans: Identification of Regulators of Morphogenesis and Fitness
Source: PLoS One. 2012 Sep 25;7(9):e45912. doi: 10.1371/journal.pone.0045912 (PMC3457969; doi:10.1371/journal.pone.0045912)

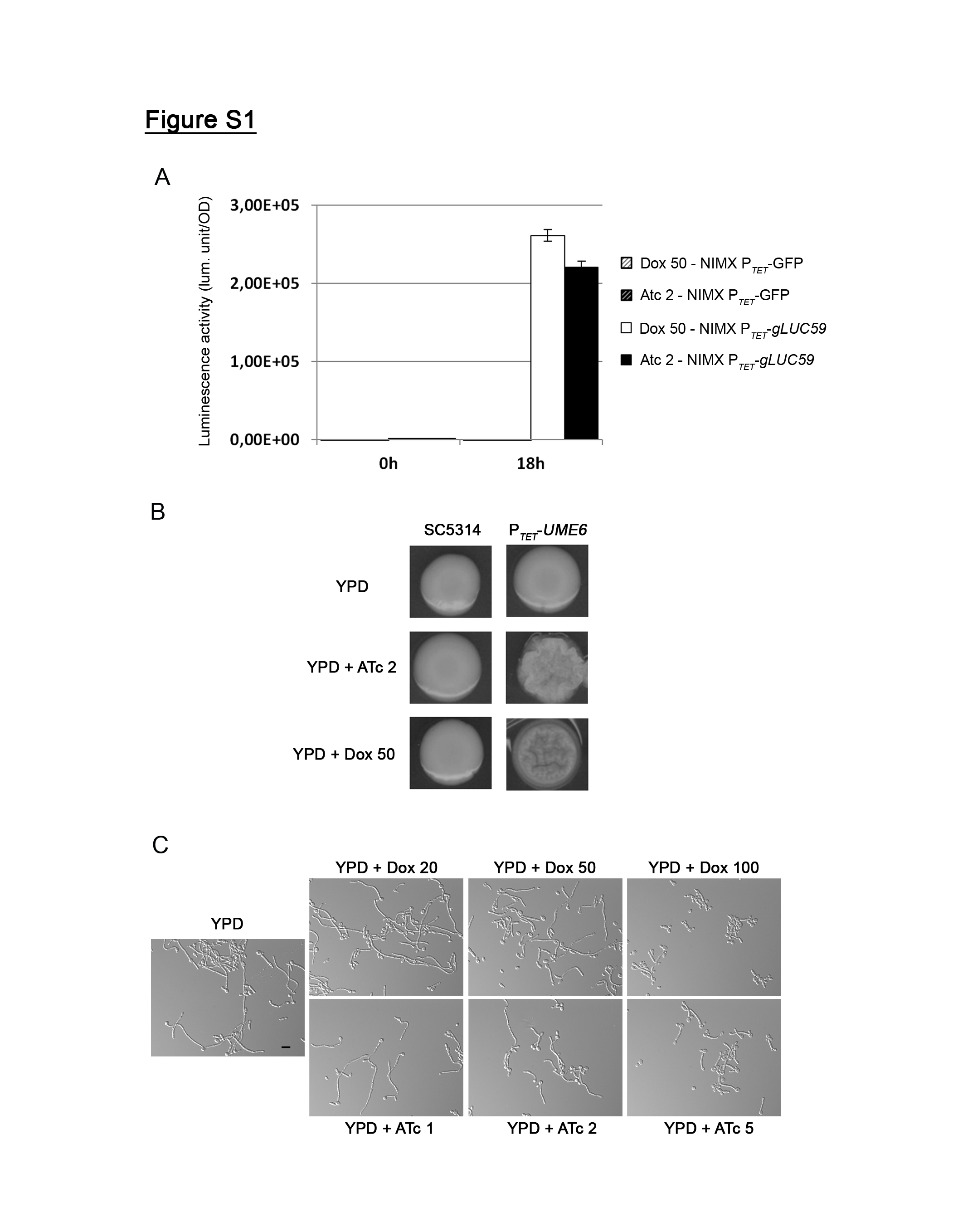

Supplement: Figure S1 — Comparison of doxycycline (Dox) and anhydrotetracycline (ATc). A. 50 µg.mL−1 Dox or 2 µg.mL−1 ATc induce P TET to a similar extent. C. albicans strains with integrated CIp10-PTET-GTW derivatives harbouring the GFP or gLUC59 ORFs (CEC2992 or CEC3083, respectively) were grown in YPD supplemented with 50 µg. mL− 1 Dox or 2 µg.mL− 1 ATc for 18 h at 30°C. Data represent luciferase specific activity detected from the different strains at 0 and 18 h of growth under inducing conditions. Assays were performed in duplicate and means and SD are shown. B. Effects on morphogenesis are similar between 50 µg.mL−1 Dox and 2 µg.mL−1 ATc. C. albicans strain SC5314 and a strain overexpressing UME6 (CEC2994) were grown in YPD medium and spotted on YPD medium supplemented or not with tetracycline analog (50 µg.mL− 1 Dox or 2 µg.mL− 1 ATc). Pictures were taken after 5 days of growth at 30°C. C. ATc shows lower inhibition of C. albicans hyphal growth than Dox. C. albicans strain SC5314 was grown in YPD liquid medium supplemented or not with different concentrations of Dox or ATc for 18 h at 30°C and observed microscopically. Scale bar = 5 µm. (TIF) [file pone.0045912.s001.tif]

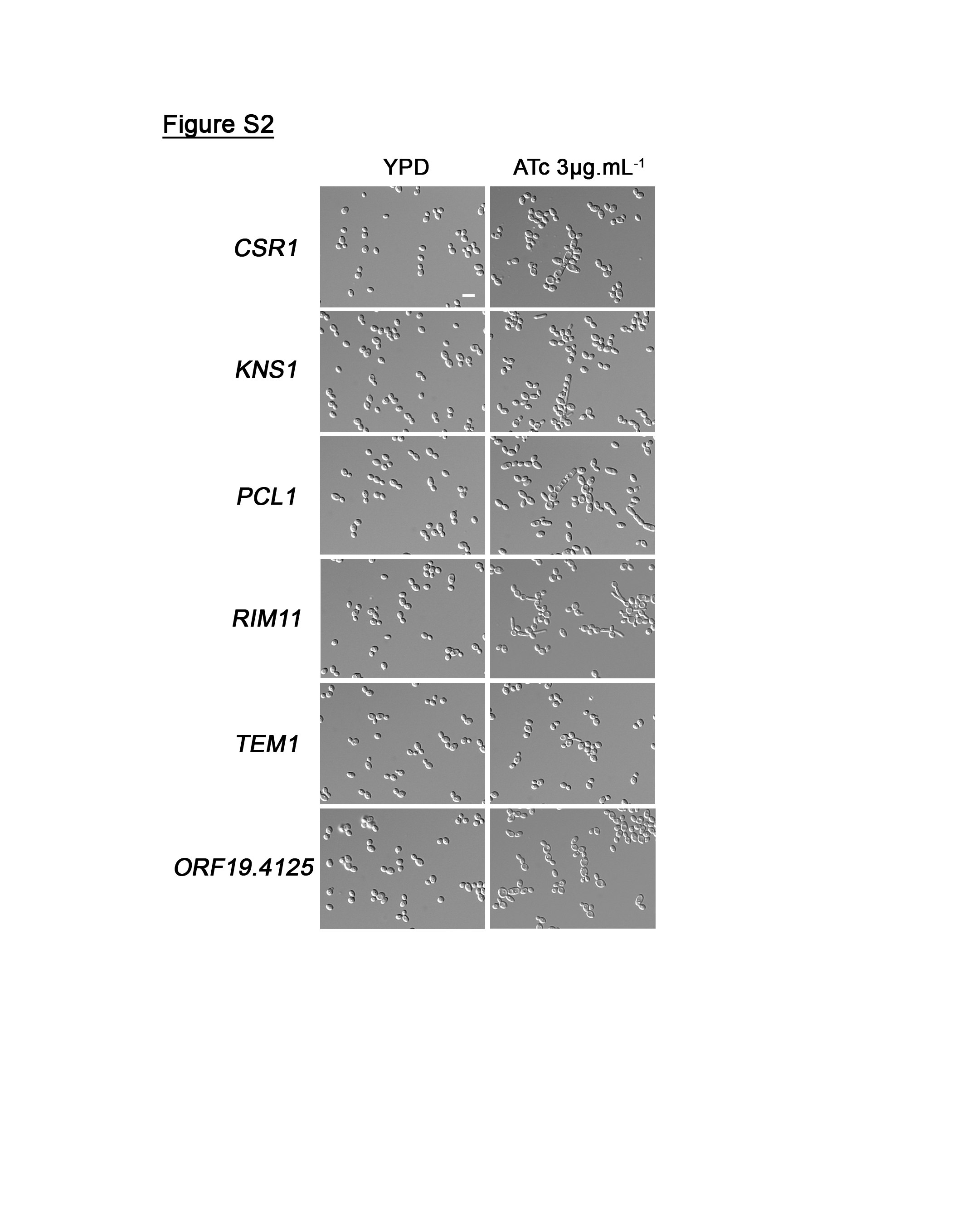

Supplement: Figure S2 — P TET -driven OE of 6 genes leads to a weak but significant phenotype in liquid media. C. albicans strains with integrated CIp10-PTET-GTW derivatives harbouring ORFs for the indicated genes were grown in YPD or YPD supplemented with 3 µg.mL− 1 ATc for 18 h. Both cultures were observed microscopically and revealed OE-associated pseudofilamentation or filamentation (germ tubes essentially). Scale bar = 5 µm. (TIF) [file pone.0045912.s002.tif]
