# Supplementary material for: A Versatile Overexpression Strategy in the Pathogenic Yeast Candida albicans: Identification of Regulators of Morphogenesis and Fitness
Source: PLoS One. 2012 Sep 25;7(9):e45912. doi: 10.1371/journal.pone.0045912 (PMC3457969; doi:10.1371/journal.pone.0045912)
Supplement: Table S2 — Candida albicans genes whose P PCK1 -driven or P TET -driven OE triggers pseudohyphal or hyphal growth. (DOCX) [file pone.0045912.s004.docx]

**Table S2: *Candida albicans* genes whose P*_PCK1_*-driven or P*_TET_*-driven OE triggers pseudohyphal or hyphal growth.**

| **ORF** | **Gene name** | **Phenotype observed in liquid / on solid medium** | **Description^1^** | | **References** |
| --- | --- | --- | --- | --- | --- |
| **Genes with a known function in filamentation** | | | |  |  |
| *ORF19.454* | *SFL1* | P*_PCK1_* : liquid P*_TET_ :* liquid / solid | Protein involved in negative regulation of flocculation and filamentous growth | | [13,56,57] |
| *ORF19.844* | *STE11* | P*_TET_* : liquid / solid | Protein similar to *S. cerevisiae* Ste11p; mutants are sensitive to growth on H_2_O_2_ medium | | [15,17] |
| *ORF19.971* | *SKN7* | P*_TET_* : liquid / solid | Putative homolog of *S. cerevisiae* Skn7p, which is predicted to be a response regulator protein in a phosphorelay signal transduction pathway; required for normal hydrogen peroxide resistance | | [67] |
| *ORF19.2649* | *PCL1* | P*_TET_* : liquid | Cyclin homolog; expression induced upon filamentous growth; transcription is induced in response to alpha pheromone in Spider medium | | [55,98,99] |
| *ORF19.2823* | *RFG1* | P*_PCK1_* : liquid P*_TET_* : liquid | Transcriptional regulator of filamentous growth and hyphal genes; acts in Tup1p-dependent and -independent pathways; not transcriptionally regulated by oxygen or serum; not responsible for hypoxic repression | | [54,55,58,100] |
| *ORF19.3207* | *CCN1* | P*_PCK1_* : liquid | G1 cyclin; required for hyphal growth maintenance not initiation; cell-cycle regulated transcription G1/S; Cdc28p-Ccn1p initiates Cdc11p S394 phosphorylation on hyphal induction | | [17,48,50,101] |
| *ORF19.3794* | *CSR1* | P*_TET_* : liquid / solid | Zinc-finger transcription factor involved in zinc homeostasis and in regulation of biofilm matrix; mutation affects filamentous growth; can suppress *S. cerevisiae* *rok1* mutant inviability | | [10,32,74,75] |
| *ORF19.3969* | *SFL2* | P*_PCK1_* : liquid P*_TET_* : liquid / solid | Probable transcription factor required for filamentous growth; required for virulence in an RHE model and in murine gastrointestinal infection but not in mouse systemic infection | | [13,53,59] |
| *ORF19.4056* | *BRG1* | P*_PCK1_* : liquid P*_TET_ :* liquid / solid | Putative DNA-binding transcription factor; similar to *S. cerevisiae* Gat2p; transposon mutation affects filamentous growth; Hap43p-repressed gene; late-stage biofilm-induced | | [10,13,17,29,60] |
| *ORF19.4433* | *CPH1* | P*_TET_* : liquid / solid | Transcription factor required for mating and hyphal growth on solid media; in filamentation pathway with Ess1p and Czf1p; required with Efg1p for host cytokine response; regulates genes of galactose metabolism | | [11,17,29,61,62,64,102] |
| *ORF19.4670* | *CAS5* | P*_PCK1_* : liquid P*_TET_* : liquid | Zinc finger transcription factor, cell wall damage response; required for transcriptional response/resistance to caspofungin; repressed in core stress response; mutants have reduced CFU in mice and hyphal defects during *C. elegans* infection | | [13,15,17,51,52,103] |
| *ORF19.5389* | *FKH2* | P*_PCK1_* : liquid P*_TET_* : liquid / solid | Forkhead transcription factor; morphogenesis regulator; required for wild-type hyphal transcription, cell separation, and for virulence in cell culture; mutant lacks true hyphae, is constitutively pseudohyphal; upregulated in RHE model | | [49] |
| *ORF19.5498* | *EFH1* | P*_TET_* : liquid / solid | Transcriptional activator; homodimer; minor role in transcriptional regulation vs Efg1p; regulates filamentous growth, phenotypic switching; *EFG1* and *EFH1* genetically interact; expression interferes with mouse intestinal tract colonization | | [66] |
| *ORF19.5908* | *TEC1* | P*_TET_* : liquid / solid | TEA/ATTS transcription factor involved in pheromone response in white cells, regulates hypha-specific genes, wild-type biofilm formation; regulates *BCR1*; transcription regulated by Cph2p in some conditions; alkaline-, and biofilm-induced | | [11,13,29,63,64,65,68,69] |
| *ORF19.6936* | *RAD53* | P*_TET_* : liquid / solid | Protein involved in regulation of DNA-damage-induced filamentous growth; putative component of cell cycle checkpoint; ortholog of *S. cerevisiae* Rad53p, protein kinase required for cell-cycle arrest in response to DNA damage | | [70] |
| *ORF19.7227* | - | P*_TET_* : solid | Putative protein phosphatase inhibitor; Hap43p-repressed; homozygous transposon insertion decreases colony wrinkling but does not block true hyphal growth in liquid media; mutation confers hypersensitivity to toxic ergosterol analog | | [16] |
| **Genes with a known function but not previously involved in morphogenesis** | | | |  |  |
| *ORF19.217* | - | P*_PCK1_* : liquid P*_TET_* : liquid / solid | Putative zinc finger protein, not required for morphogenesis | | [13] |
| *ORF19.4000* | *GRF10* | P*_PCK1_* : liquid  P*_TET_* : solid | Putative homeodomain transcription factor involved in adenine metabolism, not required for morphogenesis | | [13] |
| *ORF19.4869* | *SFU1* | P*_TET_* : solid | Transcriptional regulator of iron-responsive genes; represses iron utilization genes if iron is present; Hap43p-repressed; promotes gastrointestinal commensalism in mice | | [76,104,105] |
| *ORF19.5325* | *KIN3* | P*_TET_* : liquid / solid | Protein similar to *S. cerevisiae* Kin3p; induced under Cdc5p depletion; shows Mob2p-dependent hyphal regulation; mutants are hypersensitive to caspofungin | | [15,98] |
| *ORF19.5389* | *RIM11* | P*_TET_* : liquid | Protein not essential for viability; similar to *S. cerevisiae* Rim11p, which is a protein kinase involved in meiosis and sporulation | | [106,107] |
| *ORF19.5758* | *SAL6* | P*_PCK1_* : liquid | Putative protein phosphatase of the Type 1 family serine/threonine-specific, similar to *S. cerevisiae* Ppq1p, mutant show virulence defects | | [17,108] |
| *ORF19.7319* | *SUC1* | P*_PCK1_* : liquid | Putative transcriptional regulator with N-terminal zinc finger possibly involved in the regulation of sucrose metabolism | | [13,109] |
| **Uncharacterized genes** | | | |  |  |
| *ORF19.1577* | - | P*_TET_* : liquid / solid | Has domain(s) with predicted sequence-specific DNA binding, sequence-specific DNA binding transcription factor activity, zinc ion binding activity and role in regulation of transcription, DNA-dependent, not required for morphogenesis | | [17] |
| *ORF19.3001* | *TEM1* | P*_TET_* : liquid | Protein similar to *S. cerevisiae* Tem1p, which is a GTPase involved in termination of the M phase of the cell cycle; induced under Cdc5p depletion | | [12,14,18] |
| *ORF19.4125* | - | P*_TET_* : liquid | Putative transcription factor with zinc finger DNA-binding motif; Hap43p-induced, invasive growth decreased for heterozygous mutant in competition assay | | [11,18] |
| *ORF19.4979* | *KNS1* | P*_TET_* : liquid / solid | Ortholog(s) have protein serine/threonine kinase activity, protein tyrosine kinase activity and role in protein autophosphorylation | | [107] |

^1^Functions have been retrieved from the Candida Genome Database as of May 9, 2012 and modified according to additional literature.
