# Supplementary material for: A Versatile Overexpression Strategy in the Pathogenic Yeast Candida albicans: Identification of Regulators of Morphogenesis and Fitness
Source: PLoS One. 2012 Sep 25;7(9):e45912. doi: 10.1371/journal.pone.0045912 (PMC3457969; doi:10.1371/journal.pone.0045912)
Supplement: Table S3 — Oligonucleotides used in this study. (DOCX) [file pone.0045912.s005.docx]

**Table S3: Oligonucleotides used in this study**

| Oligonucleotide | 5’ – 3’ Sequence |
| --- | --- |
| Nco-5’Sce | CATGTAGGGATAACAGGGTAAT  I-*Sce*I |
| Nco-3’Sce | CATGATTACCCTGTTATCCCTA  I-*Sce*I |
| TAPFUF | ATCAATCATGGCGATATCAGAAAGAGAAGATGGAAAAAGA  *Eco*RV |
| TER-PVUII | GGGCAGCTGCTATGCGTCCATCTTTACAGTCC  *Pvu*II |
| TAPFUR | TCTCTTTCTGATATCGCCATGATTGATAATTATTTGAAT  *Eco*RV |
| PRPKC1PR | CGGGTACCACCACCGAGATAAGTACCCCAAC  *Kpn*I |
| TETKpn | ATAGGTACCTTTACCACTCCCTATCAGTGATAGAG  *Kpn*I |
| Tap1-PstI | TACCTGCAGGGTGCTGGCGCAGGTGCTTCCATGGAAAAGAGAAGATGG  *Pst*I |
| Tap2-AscI | ATAGGCGCGCCTCAGGTTGACTTCCCCGCGGAATTC  *Asc*I |
| TetATGE5 | TGTTGATATCGCcattgtaaattatttatatttgtatgtgtgtagg  *Eco*RV |
| Vect32 | CATAGATATCA**TGA**A**TAA**A**TAG**CATTATAAGTAAATGCATGTATAC  *Eco*RV |
| Vect33 | TTCTGATATCGCCATTGTAAATTATTTATATTTGTATGTGTGTAGG  *Eco*RV |
| Vect30 | CCTTAGGGATAACAGGGTAATAGG  I-*Sce*I |
| Vect31 | CCTATTACCCTGTTATCCCTAAGG  I-*Sce*I |
| CIpUL | ATACTACTGAAATTTCCTGACTTTC |
| CIpUR | ATTACTATTTACAATCAAAGGTGGTC |
| NIM1_verif | TTTACGGGTTGTTAAACCTTCGAT |
| ADH1_verif | ACAAGCTCATTGAGTGACGAAAAG |

U1-sac2 CCGCGGATGTCCACGAGGTCTCT

U2-sac2 CGTACGCTGCAGGTCGACCGCGG

D1-sac2 CCGCGGTGTCGGTCTCGTAG

D2-sac2 ATCGATGAATTCGAGCTCGCCGCGG
